# Fluoride-driven ‘turn on’ ESPT in the binding with a novel benzimidazole-based sensor

**DOI:** 10.3762/bjoc.11.61

**Published:** 2015-04-24

**Authors:** Kai Liu, Xiaojun Zhao, Qingxiang Liu, Jianzhong Huo, Bolin Zhu, Shihua Diao

**Affiliations:** 1 Key Laboratory of Inorganic-Organic hybrid Functional Material Chemistry, Ministry of Education, Tianjin Normal University, Tianjin, 300387, China; 2 Tianjin Key Laboratory of Structure and Performance for Functional Molecules, College of Chemistry, Tianjin Normal University, Tianjin, 300387, China

**Keywords:** anion recognition, deprotonation, ESPT, fluoride, ‘turn on’ fluorescence

## Abstract

A novel fluorescence sensor (BIP) bearing NH and OH subunits displayed a highly selective and sensitive recognition property for fluoride over other anions. Fluoride-driven ESPT, poorly used in anion recognition and sensing, was suggested to be responsible for the fluorescence enhancement with a blue shift of 35 nm in the emission spectrum.

## Introduction

Design and synthesis of selective and efficient sensors for various anions involved in biological, industrial and environmental processes have drawn a lot of attention [1–3]. In recent years, much effort has been devoted to the development of anion fluorescent sensors [4]. Of particular interest concerning anion recognition and sensing was fluoride [5–7], as it is important to human health (for example, dental care, osteoporosis and osteosarcoma). To realize the high selectivity and sensitivity to fluoride, many anion fluorescent sensors have been designed based upon numerous signal mechanisms [4,8–10]. However, excited-state intra/intermolecular proton transfer (ESPT), as an extensively exploited mechanism in many biological and chemical processes, has been employed poorly in anion recognition and sensing [2,11–16]. In the ESPT molecules, a five or six-membered intramolecular hydrogen-bonded ring formed, and a proton/hydrogen atom is transferred to an electronegative atom at the excited state [2,17–18]. By regulating the proton acidity of the H-bond donor which plays a vital role in the ESPT process, the biologically and chemically important anion would be distinguished by the different optical outputs for the interaction between sensor and anion [2,14–15,19].

During the last few years, various types of artificial sensors have been developed for anion recognition and sensing [1,4,14–15,19–20]. Among these, the hydrogen bond, especially NH [21–24], was particularly useful and effective [1,3,14–15,19–20]. However, to OH-based anion sensors it has been paid less attention [25–26]. In fact, we have succeeded in designing and synthesizing the anion fluorescent probes, based on ESPT [15–16], containing phenolic OH as H-bond donor. In these systems, the probe displayed a narrower linear range for fluoride. Furthermore, to the best of our knowledge, there were few reports on the anion sensors containing both NH and OH binding sites [14,25,27]. On the foundation of the above-mentioned background, herein we report the synthesis and H-bond donor ability of the novel anion sensor (*E*)-2-(((1*H*-benzo[*d*]imidazol-2-yl)imino)methyl)-5-(dimethylamino)phenol (BIP) made up of benzimidazole and phenol units with a C=N linker. Although BIP is weakly fluorescent, its anion-binding adduct is expected to be highly fluorescent by opening up ESPT channel [2,14–15,19]. In this paper, we demonstrated that BIP displayed selectively fluorescence enhancement for fluoride by ESPT. Importantly, BIP displayed a fluoride-selective fluorescence response with a wider linear range from 0.04 to 1.2 μM.

## Results and Discussion

**Scheme 1:**
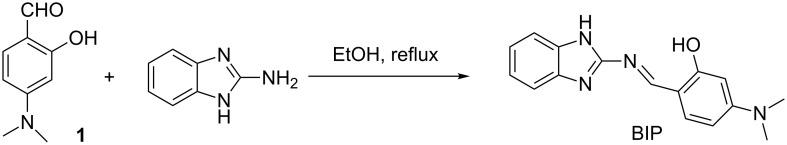
Synthesis of sensor BIP.

BIP was synthesized by the condensation of 2-aminobenzimidazole and 4-(dimethylamino)salicylaldehyde according to the literature [28–29] with little modification, as displayed in Scheme 1. It was well characterized by ^1^H NMR, mass spectrometry and elemental analysis before its practical application.

**Figure 1:**
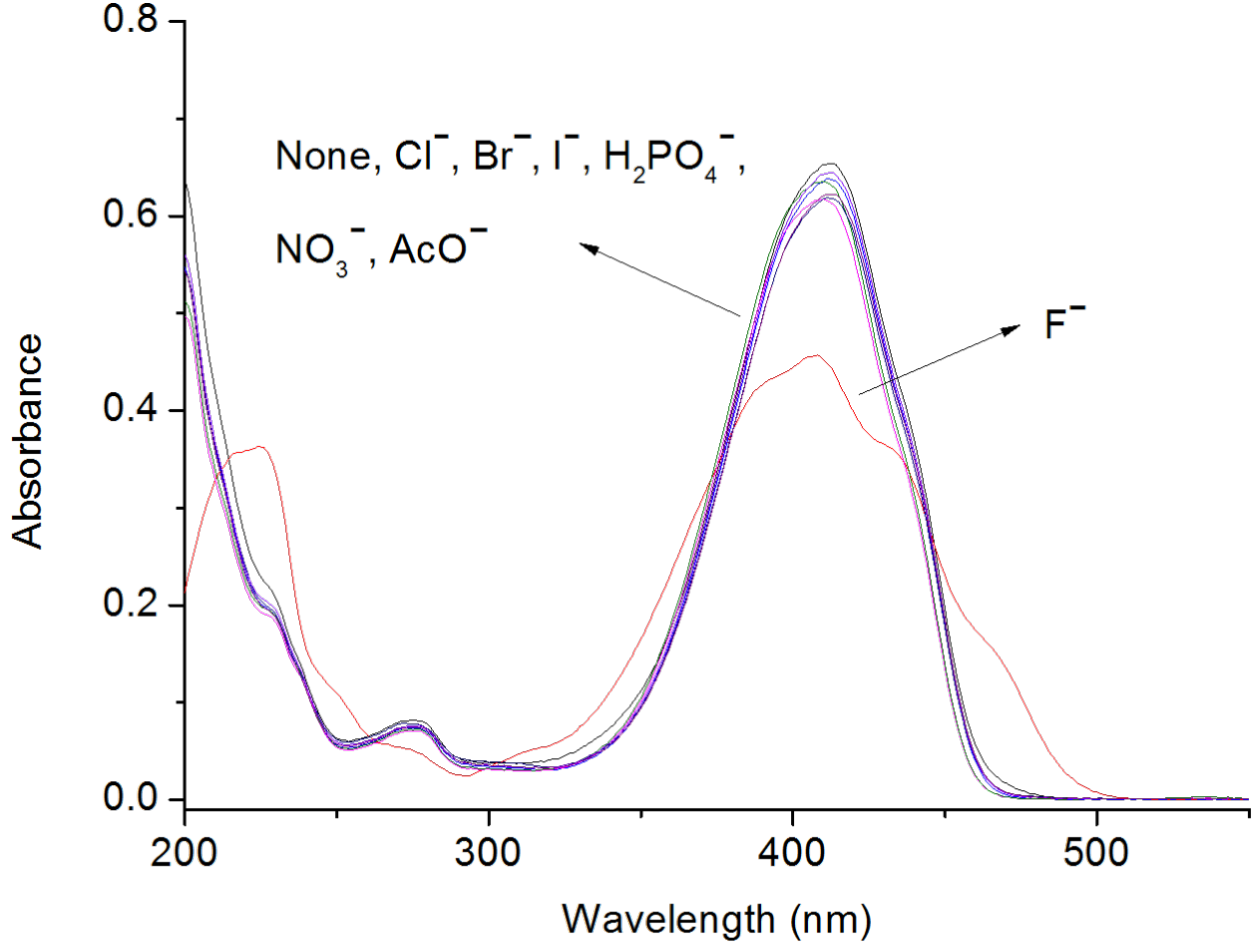
Spectral changes of BIP (4 × 10^−5^ M) in CH_3_CN after the addition of 50 equiv of anions (as their tetrabutylammonium salts).

The anion binding and sensing properties of BIP were first evaluated by UV–vis studies. There were three characteristic bands centered at 410, 276 and 228 nm in CH_3_CN. Among these, the band in the region around 276 and 228 nm is attributed to the excitation of π electrons localized at the C=N bond and at the aromatic system [30], and the peak at 410 nm was ascribed to the intramolecular charge transition between the phenol and benzimidazole [2,30]. Upon addition of F^−^ (as tetrabutylammonium salts, TBA salts), as shown in Figure 1, the intensity of the band at 410 nm decreased and broadened, and a new shoulder around 466 nm formed, meanwhile, the shoulder peak at 228 nm increased. However, no noticeable spectral changes were found for other anions (Cl^−^, Br^−^, I^−^, H_2_PO_4_^−^, NO_3_^−^, AcO^−^, added as their TBA salts). This indicated the poor interaction between BIP and these anions.

**Figure 2:**
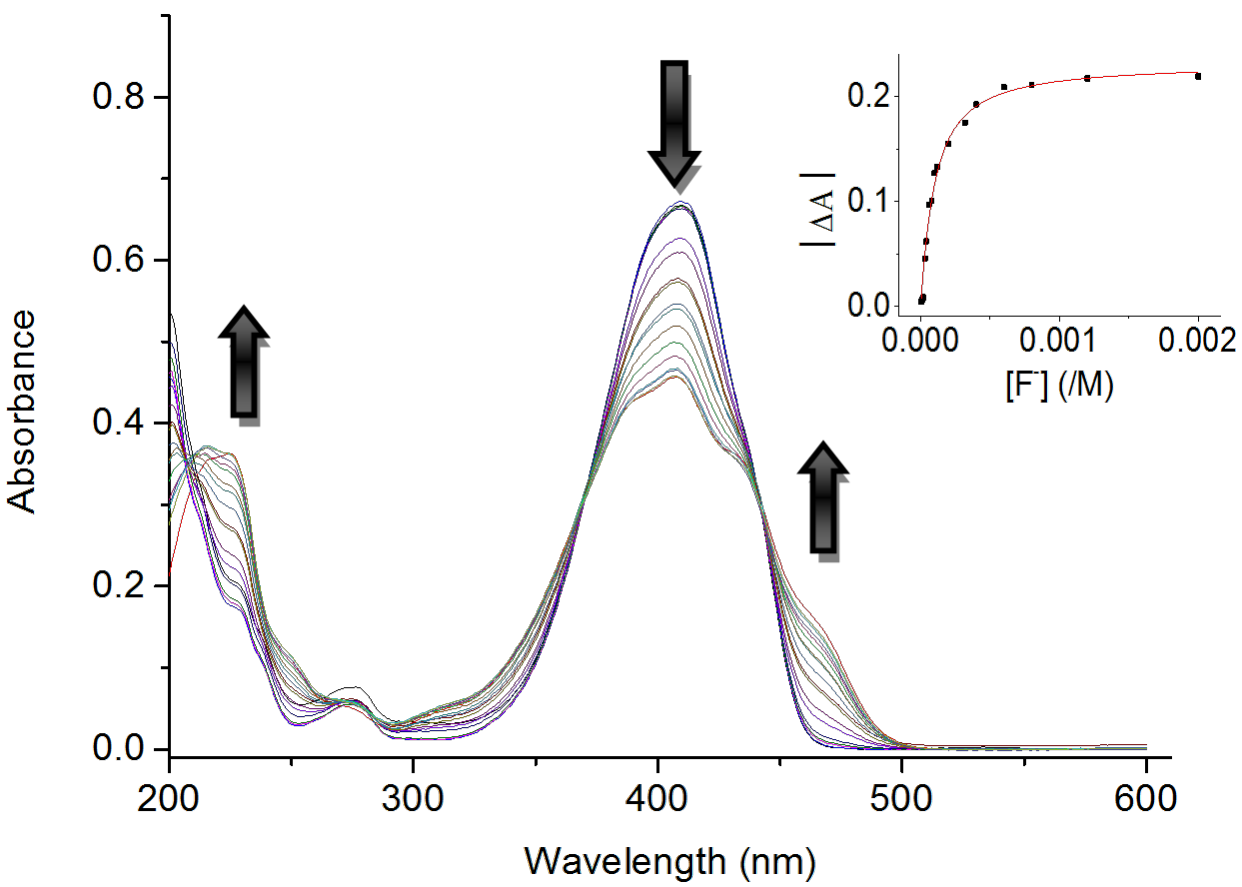
UV−vis absorption spectra of BIP (4 × 10^−5^ M) in CH_3_CN after the addition of 0–50 equiv of TBAF. Insert: Non-linear curve fit of absorbance changes at 410 nm as a function of fluoride concentration.

The interaction of BIP with fluoride was further investigated by UV–vis spectroscopic titration. As seen from Figure 2, the spectral changes with two well-defined isosbestic points (344, 270 nm) indicated the formation of a single complex between BIP and fluoride [2]. Evidence for 1:1 complex formation was also validated by non-linear least-square analysis [31] for BIP with F^−^. The association constant, calculated from the UV−vis plot at 410 nm as the function of anion concentration [15], was 1.25 × 10^4^ (*R* = 0.9926) for F^−^ (see Figure S1 in Supporting Information File 1). Due to the little spectral changes of BIP upon other anions (Cl^−^, Br^−^, I^−^, NO_3_^−^, H_2_PO_4_^−^, AcO^−^, added as TBA salts), as displayed in Figure 1, the binding constants cannot be determined. Furthermore, the similar absorption was found for BIP with F^−^ and OH^−^ (see Figure S4 in Supporting Information File 1), which suggested the occurrence of deprotonation during the titration process.

The mechanism of the UV–vis response for F^−^ was further assessed by ^1^H NMR studies, which were conducted in DMSO-*d*_6_. The characteristic peak of BIP at 12.85 ppm suggested the formation of an intramolecular hydrogen bond in BIP itself between the imine nitrogen and the phenolic OH proton [32–34], that progressively disappeared with the successive addition of F^−^ (see Figure S5 and Figure S6 in Supporting Information File 1). This indicated that the proton transfer from BIP to F^−^ [1]. Meanwhile, the NH signal of BIP at 12.41 ppm was shifted downfield, broadened and weakened, which was ascribed to the formation of an intermolecular hydrogen bond between F^−^ and the above-mentioned proton in BIP.

**Figure 3:**
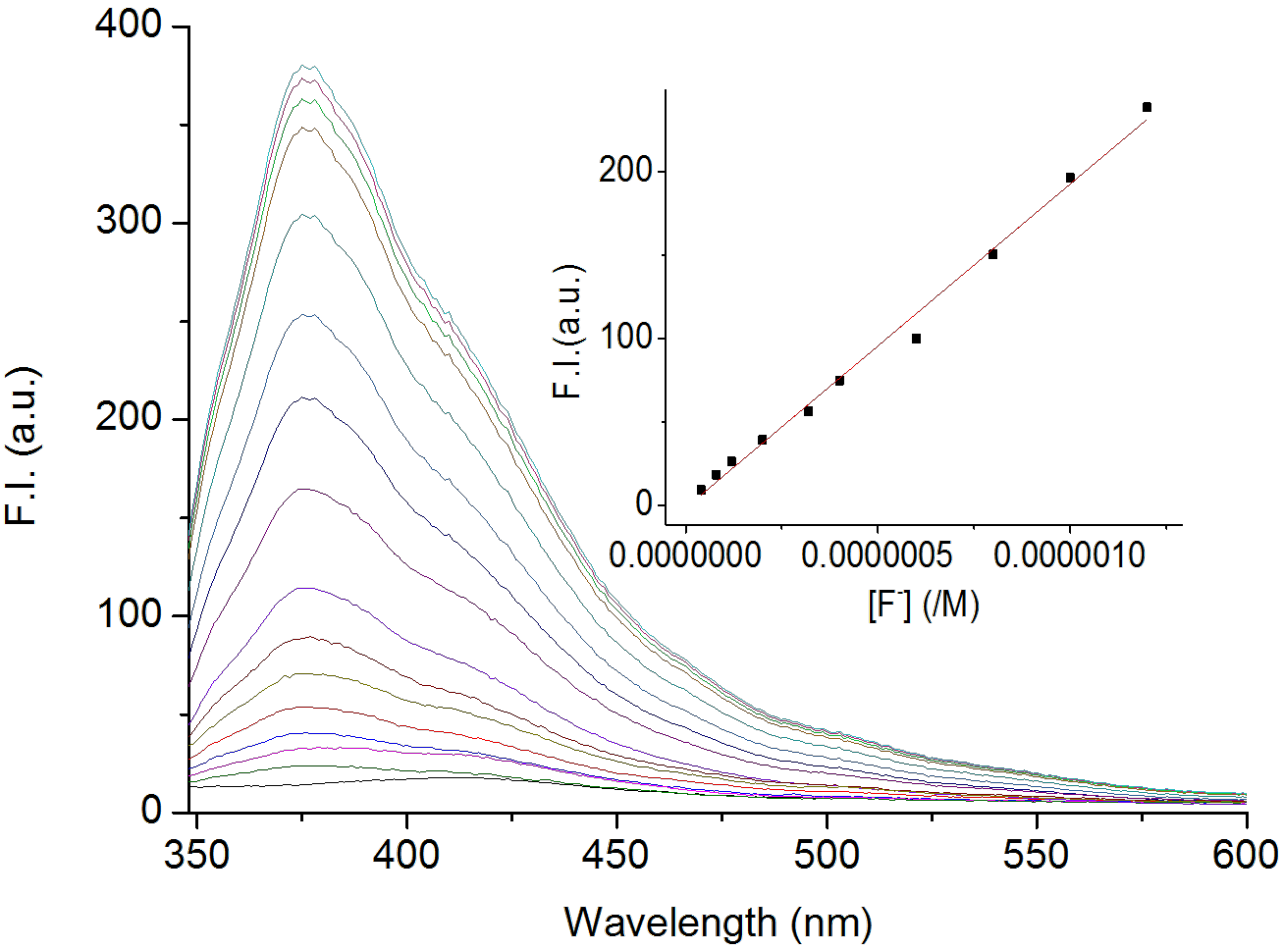
Emission spectral changes of BIP (4 × 10^−7^ M) in CH_3_CN upon addition of 0–20 equiv TBAF. Insert: Linear plot of emission intensity at 376 nm against fluoride concentration.

To further explore the photophysical sensing properties of BIP with various anions (F^−^, Cl^−^, Br^−^, I^−^, H_2_PO_4_^−^, NO_3_^−^, AcO^−^, added as TBA salts), fluorescence measurements were carried out. The weak fluorescence emission at 411 nm, corresponding to the enol form of a Schiff base [32–34], suggested the formation of the geometrically restricted six-membered intramolecular hydrogen bond ring in the BIP at transition state [32–34]. As displayed in Figure 3, with gradual addition of F^−^ to the CH_3_CN solution of BIP, the fluorescence intensity at 411 nm increased slowly. This indicated the occurrence of hydrogen-bonding interaction between BIP and F^−^. Moreover, the acidity of the OH group of BIP was expected to be enhanced by anion binding, and the ESPT channel would be open [8–10,15]. The subsequent spectral behavior proved our assumption. The new peak at 376 nm occurred and reached a limiting value after the addition of 20 equiv F^−^. The fluoride-driven ESPT displayed a fluorescence enhancement and a dramatically blue-shift with 35 nm in the emission spectrum.

**Figure 4:**
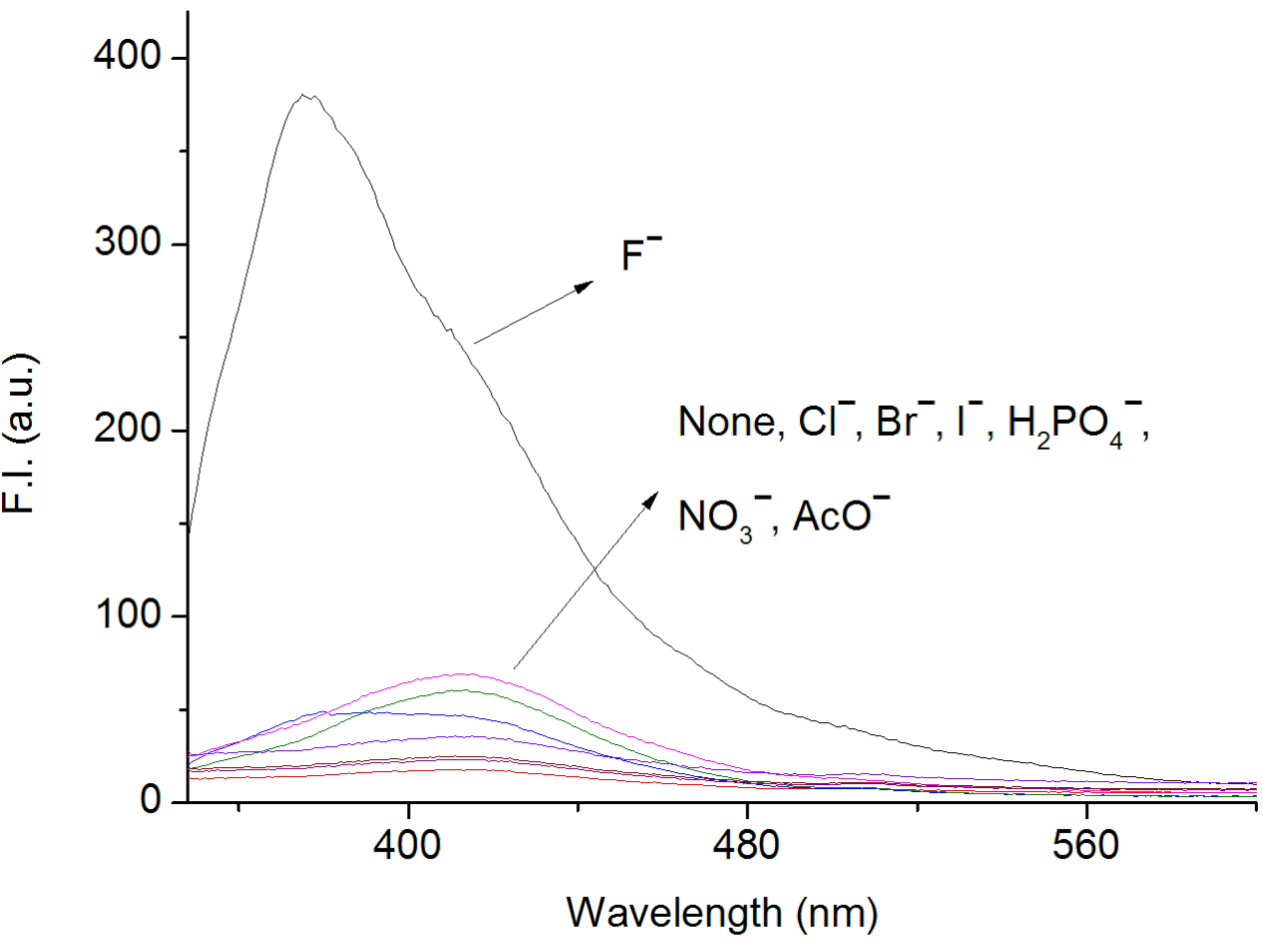
Emission spectral changes of BIP (4 × 10^−7^ M) in CH_3_CN after the addition of 20 equiv of anions (added as their TBA salts).

Analogous investigations were carried out with Cl^−^, Br^−^, I^−^, NO_3_^−^, H_2_PO_4_^−^ and AcO^−^. As shown in Figure 4, the fluorescence emission hardly experienced any perturbation on adding a similar amount of anions as done with F^−^. Also, the detection of F^−^ displayed no interference in presence of other competitive anions (See Figure S7 in Supporting Information File 1). This demonstrated BIP has a high selectivity for F^−^.

The binding constant of BIP with F^−^ was also determined by nonlinear regression based on spectrofluorometric titration. Good nonlinear curve fits (*R* = 0.9907) also indicated the formation of an 1:1 stoichiometric complex of BIP with F^−^ (see Figure S2 in Supporting Information File 1). The association constant was 8.79 × 10^5^ M^−1^, which was bigger than that obtained from UV–vis spectroscopy. This suggested the enhancement of the phenolic OH acidity by the photoexcitation [2,11–15]. Moreover, fluoride-dependent fluorescent intensity at 376 nm displayed an excellent linearity (*R* = 0.9880) in the concentration range of 0.04–1.2 μM (insert Figure 3 and Figure S3 in Supporting Information File 1). The limit of detection for F^−^ was found to be 0.021 μM, which is lower than 0.10 μM set by the US Environmental Protection Agency. This suggested that BIP had a good sensitivity for fluoride and has the potential utility to detect fluoride.

**Scheme 2:**
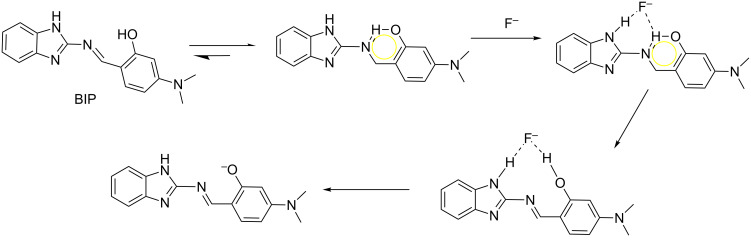
Possible binding mode between BIP and fluoride.

Based on the aforementioned UV–vis, spectrofluorimetric and ^1^H NMR studies, the possible mechanism of F^−^ recognition by BIP was proposed and illustrated in Scheme 2. On binding of F^−^, the isomerization of BIP was restricted, which enhanced the intramolecular charge transfer interaction between the assembly of binding sites with F^−^ to the chromophore [1]. With further addition of F^−^, intermolecular hydrogen bond formation disturbed the intramolecular hydrogen bonding and destroyed the geometrically restricted conformation [2], at the same time, the new geometrically restricted intermolecular H-bonding ring formed. Subsequent the fluoride-triggered ESPT occurred, which displayed ‘turn-on’ fluorescence.

## Conclusion

In conclusion, a new benzimidazole-based BIP has been developed which is selective for the recognition of F^−^ also in the presence of anions with similar basicity and surface charge density such as AcO^−^ and H_2_PO_4_^−^. Fluorescence spectral changes were driven by original hydrogen bonding interaction between BIP and F^−^, and the subsequent ESPT which resulted in the ‘turn-on’ of fluorescence. The strategy for the design of simple sensors to detect the important neutral and anionic guest may contribute to design more efficient anion chemosensors.

## Experimental

**Synthesis of compound 1:** 4-(Dimethylamino)salicylaldehyde was synthesized according to the literature [15–16] with little modification. POCl_3_ (4.0 mL, 43.2 mmol) was added dropwise to dry DMF (21.0 mL, 271.8 mmol) containing 3-(dimethylamino)phenol (3.10 g, 22.6 mmol) at 0 °C, and the mixture was stirred for 10 min, slowly warmed to room temperature and stirred for another 30 min. The reaction mixture was heated at 80 °C overnight. After cooling to room temperature, the mixture was poured into ice cold water. The solution was neutralized with saturated Na_2_CO_3_. The precipitate was washed several times with distilled water, and dried under vacuum to yield 3.30 g of **1**. ^1^H NMR (400 MHz, CDCl_3_) δ 3.09 (s, 6H, CH_3_), 6.10–7.29 (m, 3H, Ar-H), 9.53 (s, 1H, OH), 11.61 (s, 1H, CHO). This was consistent with the literature.

**Synthesis of compound BIP:** 2-Aminobenzimidazole (0.80 g, 6 mmol) and compound **1** (0.99 g, 6 mmol) were dissolved in 25 mL ethanol. The resulting solution was heated at reflux for 4 h. The solvent was removed by a rotary evaporator at reduced pressure. The crude product was purified by silica gel column chromatography (ethyl acetate/*n*-hexane 1:3) and recrystallized with ethanol to yield yellow solid. Yield: 1.03 g (61.2%); ^1^H NMR (400 MHz, DMSO-*d*_6_) δ 3.05 (s, 6H, CH_3_), 6.16–7.53 (m, 7H, Ar-H), 9.34 (s, 1H, CH=N), 12.41 (s, 1H, NH), 12.85 (s, 1H, OH); ESIMS: 280.1408 (M + H^+^).

## Supporting Information

File 1 Data analysis of QP after the addition of fluoride.
