# Supplementary material for: Fluoride-driven ‘turn on’ ESPT in the binding with a novel benzimidazole-based sensor
Source: Beilstein J Org Chem. 2015 Apr 24;11:563–7. doi: 10.3762/bjoc.11.61 (PMC4419554; doi:10.3762/bjoc.11.61)
Supplement: File 1 — Data analysis of QP after the addition of fluoride. [file Beilstein_J_Org_Chem-11-563-s001.pdf]

Supporting Information

for

Fluoride-driven ‘turn on’ ESPT in the binding with a novel benzimidazole-based sensor

Kai Liu*[1,2], Xiaojun Zhao[1], Qingxiang Liu[2], Jianzhong Huo[2], Bolin Zhu[2], Shihua Diao[2]

Address: [1]Key Laboratory of Inorganic-Organic hybrid Functional Material Chemistry, Ministry of Education, Tianjin Normal University, Tianjin, 300387, China and [2]Tianjin Key Laboratory of Structure and Performance for Functional Molecules, College of Chemistry, Tianjin Normal University, Tianjin, 300387, China

Email: Kai Liu* - hxxylk@mail.tjnu.edu.cn

*Corresponding author

1. Binding constants determination by UV–vis and fluorescence methods

Binding constants (K) were calculated by non-linear least-square analysis of the titration curves according to the following relation for 1:1 complexation.

$$\Delta X = A\left\{C_H + C_G + B - \left[\left(C_H + C_G + B\right)^2 - 4C_H C_G\right]^{\frac{1}{2}}\right\}$$

where ΔX is the difference value between the absorbance (or emission intensity) of the whole system and the absorbance of free host, A is a floating parameter in the analysis, B is the ratio between 1 to k (binding association), C_H and C_G are the concentration of host and guest, respectively.

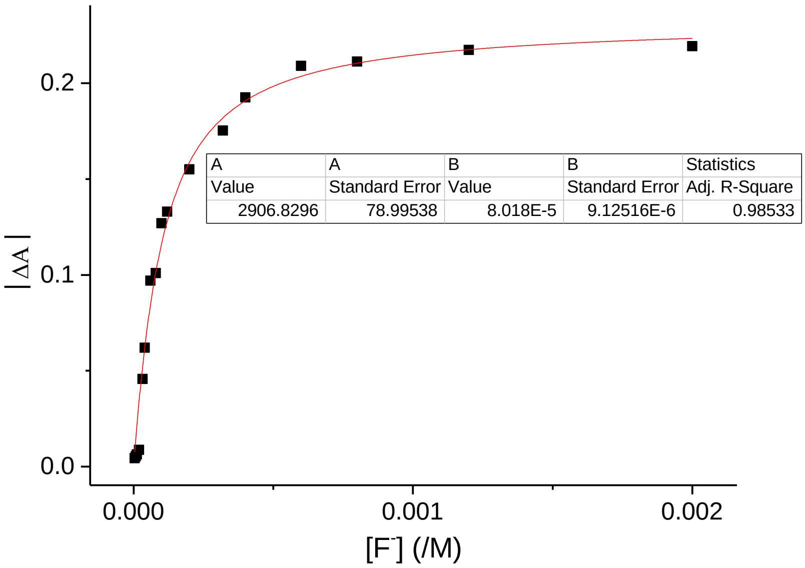

Figure S1: Non-linear curve fit of absorbance changes at 410 nm as a function of fluoride concentration.

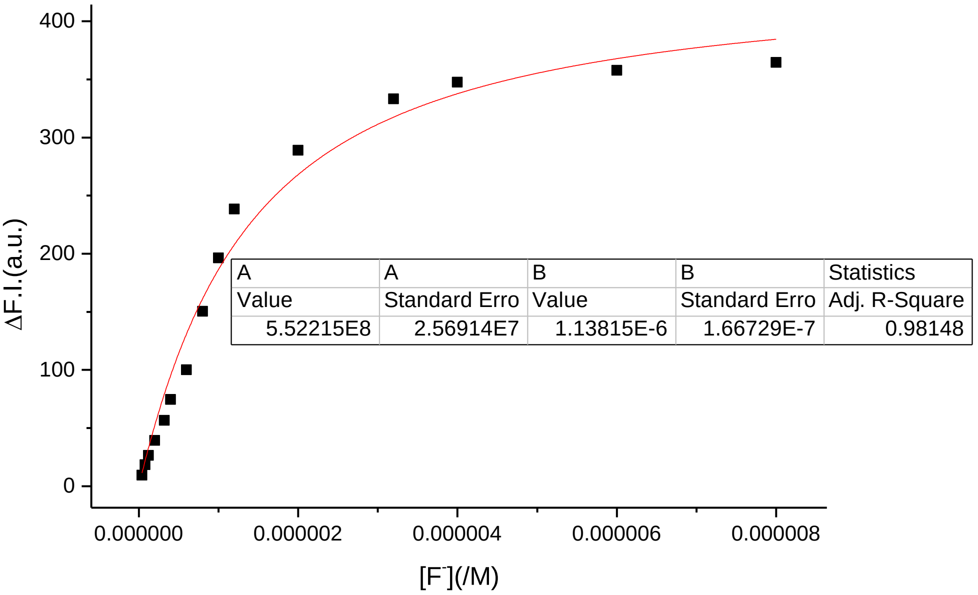

Figure S2: Non-linear curve fit of emission intensity at 376 nm as a function of fluoride concentration.

2. Calculation of detection limit

The detection limit (D) of BIP in emission spectra for fluoride ion was determined according to the followed equation:

$$D = 3S_b / k$$

Where S_b is the standard deviation of the blank solution; k is the slope of the curve.

From the Figure S3, we get the slope, which was 156553000. And the standard deviation S_b was 1.1166. Then the detection limit was obtained to be 0.021μM using the above-mentioned equation.

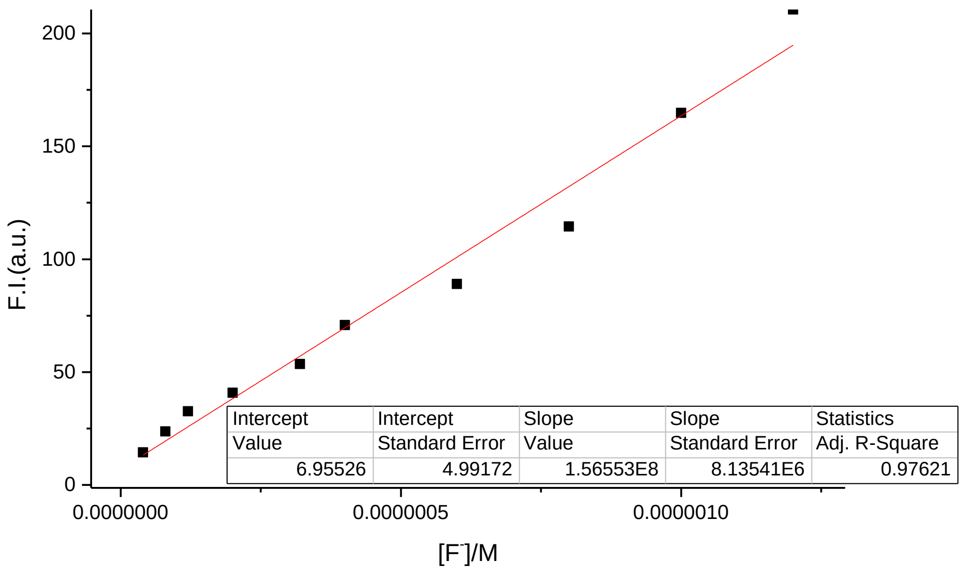

Figure S3: Changes of fluorescence intensity of BIP at 376 nm as a function of fluoride concentration.

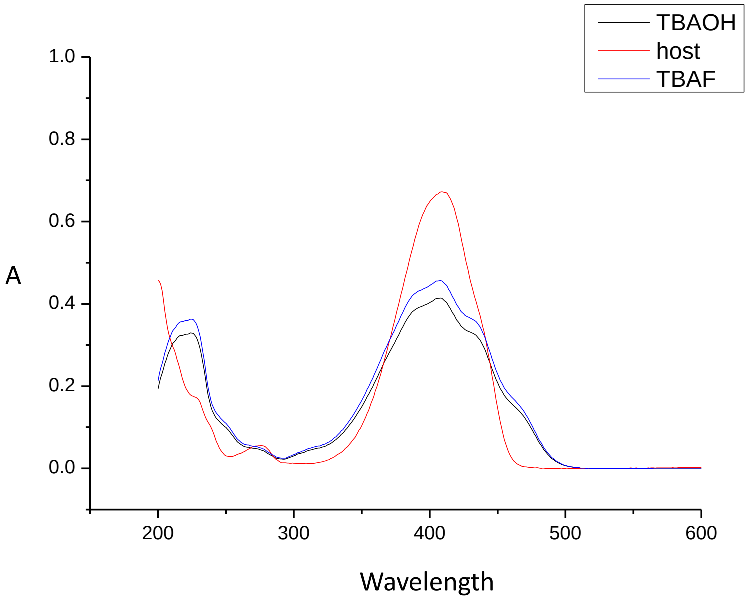

Figure S4: Spectral changes of BIP upon addition of F^- and OH^-.

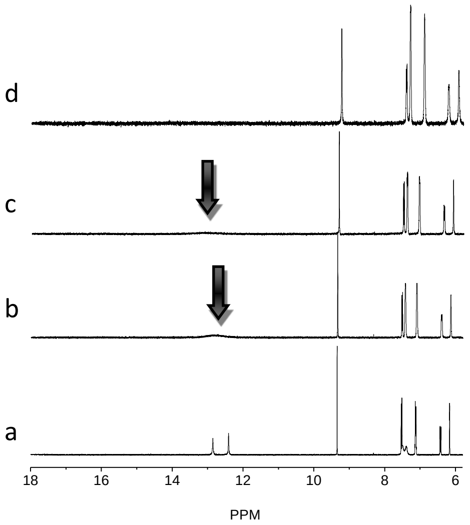

Figure S5: Partial ^{1}H NMR spectra of BIP in DMSO-d_6 upon the addition of F^-: (a) 0 equiv; (b) 0.5 equiv; (c) 2.0 equiv; (d) 5.0 equiv. Arrow marked the NH signal.

a

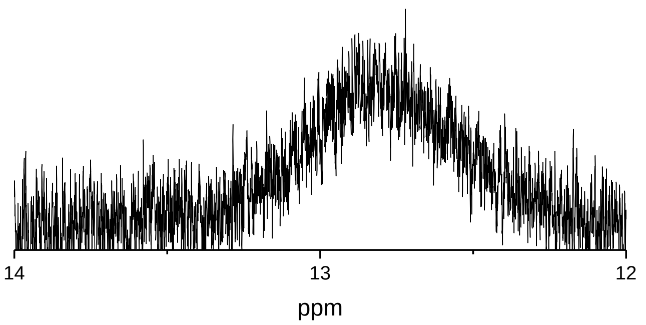

b

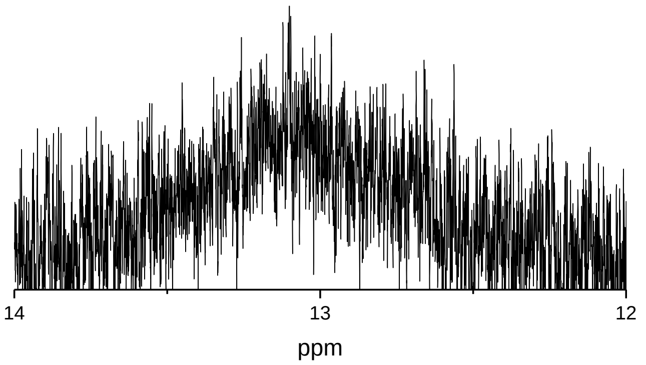

Figure S6: Partial ^{1}H NMR spectra of BIP in DMSO-d_6 upon the addition of F^{-}: (a) 0.5 equiv.; (b) 2.0 equiv.

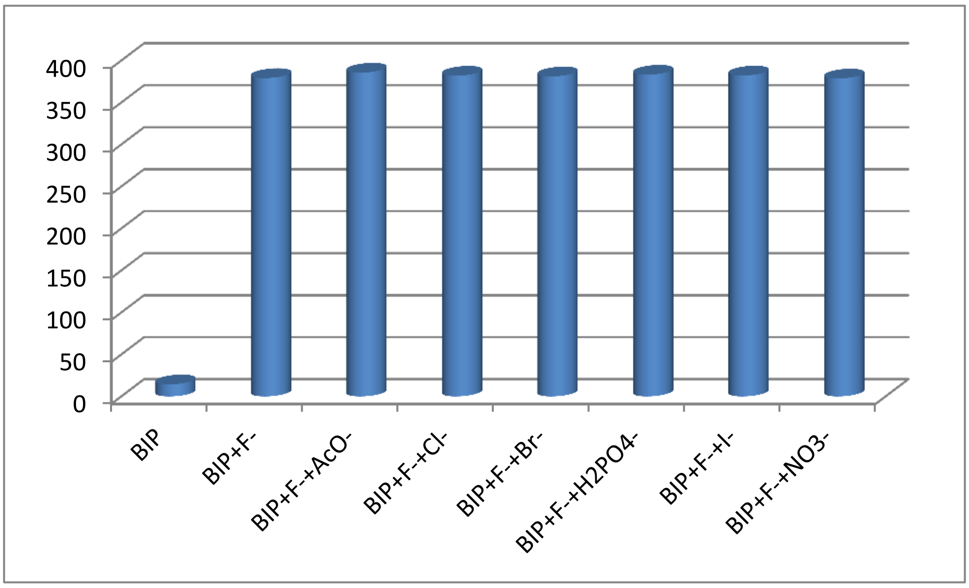

Figure S7: Emission spectral changes of BIP (4.0×10^{-7} mol/l) containing 20.0 equiv fluoride upon the addition of 20.0 equiv other anions (Cl^-, Br^-, I^-, $H_2PO_4^-$, NO_3^- and AcO^-).
